# Novel Biocomposite Films Based on High Methoxyl Pectin Reinforced with Zeolite Y for Food Packaging Applications

**DOI:** 10.3390/foods11030360

**Published:** 2022-01-26

**Authors:** Aleksandra Nesic, Sladjana Meseldzija, Gustavo Cabrera-Barjas, Antonije Onjia

**Affiliations:** 1 Department of Chemical Dynamics and Permanent Education, Vinca Institute of Nuclear Sciences—National Institute of the Republic of Serbia, University of Belgrade, Mike Petrovica-Alasa 12-14, 11000 Belgrade, Serbia; sladja_ms@yahoo.com; 2 Unidad de Desarrollo Tecnológico (UDT), Universidad de Concepción, Av. Cordillera 2634, Parque Industrial Coronel, BioBio, Concepción 3349001, Chile; g.cabrera@udt.cl; 3 Faculty of Technology and Metallurgy, University of Belgrade, Karnegijeva 4, 11120 Belgrade, Serbia; onjia@tmf.bg.ac.rs

**Keywords:** high methoxyl pectin, zeolite Y, biopolymers, strawberry shelf-life, polysaccharides

## Abstract

Pectin is a natural biopolymer with broad applications in the food industry and it is suitable to prepare edible films to prolong food shelf-life. However, the main limitation of pectin-based films is their poor mechanical and barrier properties. Zeolite Y is a hydrophobic clay that can be used as film reinforcement material to improve its physicochemical and mechanical properties. In this work, the influence of high methoxyl citrus and apple pectin on physicochemical properties of biopolymer films modified with zeolite Y (0.05–0.2 wt%) was investigated. The films were characterized by FTIR, TGA, WAXD, mechanical analysis, and water vapor permeability analysis, and a potential film application is presented. The WAXD and FTIR analysis demonstrated that the strongest interaction between pectin chains and zeolite Y occurred when citrus high methylated pectin was used. Adding 0.2 wt% of zeolite Y into citrus high methylated pectin matrix enhanced the tensile strength by 66%, thermal stability by 13%, and water vapor barrier by 54%. In addition, fruit shelf-life test was performed, where strawberries were sealed in film. It was shown that sealed strawberries maintained a better color and healthy appearance than the control treatment after 7 days at 10 °C. This study enabled the development of biocomposite films with improved properties for potential application in food packaging.

## 1. Introduction

Polymers have become increasingly dominant in the consumer marketplace for being relatively inexpensive and offering great physicochemical properties in terms of thermal, mechanical, and moisture resistance. Global plastic production and consumption are estimated at 367 million metric tons per year [1]. Among all applications, food and beverage packages are the largest application segment. The most commonly used polymers for packages, namely polyethylene and polypropylene, are derived from fossil fuels and require a long time for complete degradation [2]. This points to the scale of a particular environmental problem, which is an increasing amount of non-biodegradable plastic waste and its accumulation in nature. Thus, there is an urgent and compelling need to develop environmentally friendly biodegradable polymers as an alternative to fuel-based plastics. The most frequently investigated biopolymers are polysaccharides, i.e., chitosan, cellulose, starch, pectin, and alginate, owing to their biodegradability, nontoxicity, wide abundance, and great film-forming properties.

Pectin has a very complex structure that is not completely known yet. It is composed of α-1,4-linked d-galacturonic acid units interrupted by neutral sugar residues, particularly arabinose, galactose, and rhamnose [3]. The remaining neutral sugars are mainly present as sidechains, forming “hairy” regions. The total content of neutral sugars varies with source and extraction conditions. In nature, some pectin carboxylic groups are in a methyl ester form. The ratio of esterified to total galacturonic acid units represents an esterification degree. Pectins with more than half of carboxylic groups in a methyl ester form are classified as high methylated ones (HM). In contrast, those containing less than half are classified as low methylated (LM). Esterification and the origin of pectin and its molecular weight strongly affect solubility, viscosity, and gelation properties, thus also influencing physicochemical properties of materials [4]. Up to date, pectins are commonly used in the food industry as stabilizers and thickeners thanks to their ability to gel in sugar and acidic conditions [5,6]. Their biocompatibility offers a potential application for drug delivery systems [7,8] and biomedicine [9].

Moreover, pectins belong to a group of nontoxic and food grade biopolymers, which makes them suitable for preparation of edible coatings to prolong shelf-life of food products [10]. Moreover, pectin biocomposite films can be prepared for the same purpose; however, their main limitations are their low mechanical properties and moisture resistance. The introduction of inorganic fillers into biopolymer matrix could overcome these drawbacks. Large biopolymer matrix/filler interfacial area occurs when the latter is uniformly distributed in the polymer matrix. This leads to changes in molecular mobility, relaxation behavior, as well as mechanical and thermal stability of bio-composite materials. Only few studies on pectin-based composite films have been reported. Among inorganic fillers, montmorillonite [11,12,13,14], halloysite nanotubes [15], laponite [16], and hydroxyapatite [17] were investigated as reinforcement for pectin-based materials.

In this work, hydrophobic zeolite Y (ZY), composed of a tridimensional channel system that can be accessed through a 12-member ring (pore dimeter = 7.4 Ȧ), was incorporated into the pectin matrix. Zeolite Y belongs to a family of aluminosilicate molecular sieves with a faujasite-type structure (FAU), which is characterized by the basic formula|(Ca, Mg, Na_2_)_29_ (H_2_O)_240_|[Al_58_ Si_134_ O_384_]—FAU. It is one of the most industrially employed zeolites, owing to its unique structure, which allowed efficient use as catalyst support, in membrane separations, and as a gases absorbent [18]. The influence of ZY various concentrations on structural and physicochemical properties of biocomposite films was evaluated. Moreover, polyglycerol was used as a plasticizer, because it was demonstrated before that longer molecular chains of the plasticizer could provide stronger intermolecular bonds with the pectin matrix [19]. It was hypothesized that the introduction of hydrophobic additive and long-chain plasticizer in the pectin matrix could improve the mechanical properties and influence the barrier properties of the films. In order to estimate the most suitable film formulation that can meet criteria for food packaging application, two different types of pectins were tested: high methylated citrus pectin and high methylated apple pectin. Finally, the best film formulation would be used to test the visual appearance and weight loss of strawberries stored at 7 °C within 10 days. To the best of our knowledge, there is no research on the reinforcing effect of ZY and its influence on the mechanical, thermal, and barrier properties of polymeric films composed of pectins originating from various renewable sources, and its test in real time food packaging application.

## 2. Materials and Methods

### 2.1. Materials

High methylated citrus pectin, with a degree of esterification of 68% and molecular weight of 70,000 g/mol, was supplied by Herbstreith Fox, Germany. The second pectin type, namely high methylated apple pectin with a degree of esterification of 70% and molecular weight of 75,000 g/mol, was supplied by Sigma Aldrich, Switzerland. Polyglycerol (PG), a mixture containing a minimum 85% of diglycerol, triglycerol, tetraglycerol, and only traces of glycerol, was purchased from Solvay, Belgium. Zeolite Y was received as a gift from the Institute of General and Physical Chemistry in Belgrade. Its major physicochemical properties are presented in Table 1.

### 2.2. Preparation of Composite Films

Two different solutions of pectin (citrus high methylated pectin (PC) and apple high methylated pectin (PA)) were prepared by dissolving pectin (2 *w/v*) in distilled water at room temperature. After complete dissolution, polyglycerol (30 wt% per mass of pectin) used as a plasticizer was added to enhance film flexibility. Film-forming solutions (50 mL each) were cast into Petri dishes with a diameter of 110 mm and dried at room temperature for 24 h. Afterward, the films were stored in desiccators containing saturated magnesium nitrate solution at 25 °C and 53% relative humidity until use.

A dispersion with 1 wt% of zeolite in deionized water was homogenized in an ultrasound bath for 15 min at 50% amplitude. Dispersions at different concentrations were added to pectin/polyglycerol solutions and mixed for 6 h. The content of ZY in film-forming solutions was 0.05, 0.1, and 0.2 wt%, whereas the concentrations of pectin (2 wt%) and polyglycerol (30 wt%) were kept constant. The resulting solutions were cast into Petri dishes and stored as described above. The compositions are reported in Table 2.

### 2.3. Biocomposites’ Characterization

#### 2.3.1. FTIR-ATR Analysis

Fourier transform infrared spectroscopy spectra in attenuated total reflectance mode (FTIR-ATR) were collected in transmission mode using a Perkin Elmer Spectrum 100 spectrometer (Waltham, MA, USA) in the range of 4000–400 cm^−1^ at a resolution of 4 cm^−1^ and 64 scans per sample.

#### 2.3.2. WAXD Analysis

Wide-angle X-ray diffraction (WAXD) data were collected with a Philips PW 1050 diffractometer and Cu-Kα 1.2 radiation (λ = 1.54059 Ȧ) at room temperature. Measurements were done in the 2θ range of 6–70° with a scanning step of 0.05° and 4 s/step.

#### 2.3.3. Water Vapor Permeability (WVP)

WVP was determined gravimetrically according to the ASTM E96 standard. Films were sealed in a 35 mm circular opening of a steel permeation cell containing distilled water (∼100% relative humidity inside the cell). The permeation cell was kept in a chamber with controlled relative humidity of 50%. Weight of the permeation cell was measured every 2 h until a constant weight was reached. WVP [g/m Pa s] was calculated using the following Equation (1):

(1) $$\mathrm{WVP}=\frac{\Delta G\times l}{t\times A\times \Delta p}$$

where ΔG is weight change [g], t is time during which ΔG occurred [h], A is testing cup area [m^2^], l is film thickness [m], and Δp is water pressure difference between both sides of a film [Pa]. Measurements were performed three times and an average value was calculated. The standard deviation of WVP values was ±5%.

#### 2.3.4. Mechanical Properties

The mechanical properties of biocomposites, such as tensile strength (TS, MPa), Young’s modulus (E, MPa), and elongation at break (ε, %), were measured with an Instron M 1185 universal testing machine. The crosshead speed was 2 mm min^−1^ for all tested samples. The reported mechanical parameters are average values calculated from twelve measurements. The obtained measurement error values of tensile strength and elongation at break were within ±5%, while those of the Young’s modulus fluctuated in the range of ±10%.

#### 2.3.5. Thermogravimetric Analysis

Thermal stability of composite films was conducted by thermogravimetric analyzer Netzsch TG 209 F1 Libra^®^. The measurements were carried out in the temperature range between 25 and 600 °C at a heating rate of 10 °C/min under nitrogen flow. Approximately 5 mg samples were placed in ceramic pans. Decomposition temperatures were determined at 100 °C, 180 °C, and 350 °C, as well as main degradation steps.

#### 2.3.6. Statistical Analysis

The presented results are mean values of independent experiments with ± standard deviations. One-way analysis of variance (ANOVA) followed by Tukey’s test was used to compare mean values. For mechanical and water vapor barrier results, differences were considered significant at *p* < 0.05.

## 3. Results

### 3.1. FTIR-ATR Analysis

**Figure 1:**
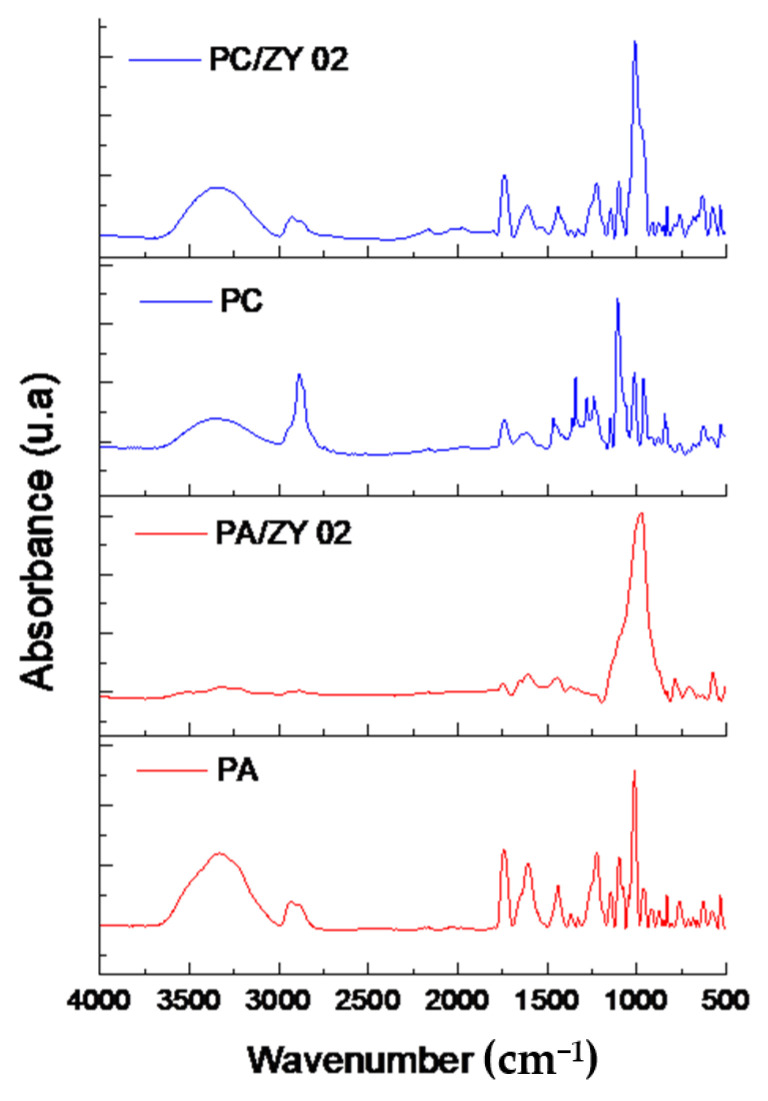
FTIR-ATR spectra of pure pectin films (PC and PA) and their biocomposite films containing Zeolite Y clay.

FTIR spectra of the pectin-based films are presented in Figure 1. In spectra of pure pectin films (PA and PC), several characteristic bands were detected: broadband in the range of 3600 and 3000 cm^−1^ ascribed to OH stretching vibrational modes of inter- and intramolecular hydrogen bonding of the galacturonic acid; moderately intense bands between 3000 and 2500 cm^−1^, related to CH, CH_2_, and CH_3_ stretching and bending vibrations; intense absorption bands of around 1740 cm^−1^ and 1600 cm^−1^, attributed to ester carbonyl (COCH_3_) groups and asymmetrical stretching band of carboxylate ion (COO^−^); less intense band at 1400 cm^−1^ referring to COO^−^ symmetric stretching vibrations; and bands in the range of 1380 cm^−1^ and 800 cm^−1^ assigned to stretching vibrations of C-C and C-O-C of the carbohydrate ring [20,21].

ZY characteristic peaks were found at 1050 cm^−1^ and 706 cm^−1^ (asymmetric and symmetric stretching vibrations of inner Si/Al-O_4_ structure, respectively), 1180 cm^−1^ and 788 cm^−1^ (asymmetric and symmetric stretching vibrations of external Si/Al-O_4_ structure, respectively), 576 cm^−1^ (vibrations of double-ring external linkage), and 455 cm^−1^ (bending vibrations of Si/Al-O_4_ structure) [22]. A zeolite addition into pectin matrix caused bands’ widening in the 1150–1000 cm^−1^ region due to overlapping of stretching vibrations of functional Si/Al-O_4_ and stretching vibrations of C-C/C-O-C pectin bonds. In all FTIR spectra of composite films, additional peaks at 455 cm^−1^ and 576 cm^−1^ appeared as a result of zeolite presence in the films. Moreover, in the case of PC and PA composite films, it is worth underlining that a zeolite addition shifted -OH band towards lower values of frequency. Moreover, the intensity of this band decreased with an increasing zeolite content. These changes were more remarkable in the case of PC composite films suggesting stronger interaction of citrus pectin with zeolite, in comparison with apple pectin.

### 3.2. Wide-Angle X-ray Diffraction (WAXD)

**Figure 2:**
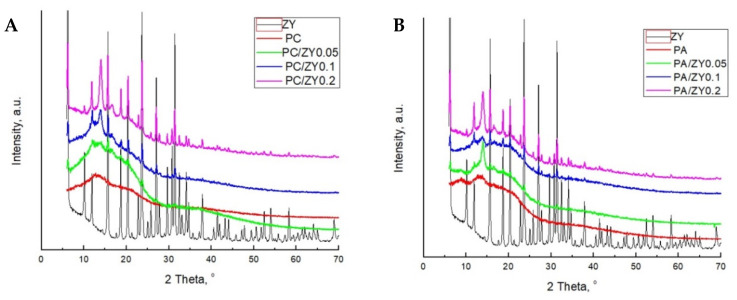
WAXD diffractograms of biocomposite films based on (**A**) citrus pectin and (**B**) apple pectin loaded with zeolite Y clay.

Wide-angle X-ray diffraction (WAXD) is an effective method to verify whether an intercalated or exfoliated composite structure was obtained. Intercalated composites are in principle structured polymeric layers inserted into clay galleries. The formation of the intercalated composite may be verified by an increase in basal spacing in WAXD patterns, which causes a shift of diffraction peak towards lower angle values. In the case of exfoliated composites, individual clay layers are separated in a continuous polymer matrix with average distances depending on the clay amount. This in turn leads to the formation of disordered structure and the absence of peaks corresponding to clay in composite materials [23,24]. WAXD profiles of pectin films, zeolite, and composite films are shown in Figure 2.

Typical zeolite peaks at 2θ = 6.25, 10.15, 11.90, 15.65, 18.70, 20.30, 23.65, 27.05, 31.40, 34.00, and 37.90 were identified. The diffraction peaks match well with the zeolite Y structure standardized by International Zeolite Association (JCPDS 43-0168) [25]. All pure pectin films showed two broad halos at 2θ = 10–13 and 20–22.

Upon the filler addition, peaks at 2θ = 15.65, 18.70, 20.30, 23.65, 27.05, and 31.4 appeared. The formation of composites was evidenced by different WAXD patterns in the composite films in comparison with raw materials, which resulted from the interactions between pectin polar groups and zeolite. The WAXD pattern of the composite films that contained 0.05 and 0.1 wt% of zeolite revealed a lower degree of crystallinity with respect to the composite films with 0.2 wt% of zeolite. However, from the perfect concordance of the positions of the reflections in the X-ray diffraction patterns, it could be concluded that all composites are likely to have the same crystalline form. This result implied partial exfoliation/intercalation of pectin chains into zeolite galleries. Among all tested films, the highest intensity of peaks was observed for PC/Z20 film, hence high methylated citrus pectin was more compatible with zeolite in comparison with apple and low methylated amidated pectins.

It is worth emphasizing that two additional diffraction peaks, located around 13.5 and 16.5, appeared in the case of all composite samples that were not present in zeolite or pectin diffractograms. This is due to an interfacial crystallization effect favored when the pectin matrix is plasticized with polyglycerol. The crystallization effect was more pronounced for PC composite films than for PA films.

### 3.3. Water Vapor Permeability (WVP)

One of the main functions of food packages is to avoid or at least minimize moisture transfer between food and atmosphere. Hence, water vapor permeability parameter should be maintained as low as possible. The WVP values of pectin-based composite films in functions of pectin type and filler concentration are shown in Table 3.

The WVP values of the PC and PA were 4.47 × 10^−10^ and 4.45 × 10^−10^ g/msPa, respectively. Negligible differences between WVP values for pure citrus and apple pectin films were obtained as they revealed approximately similar esterification degrees. In all cases, the filler addition reduced the permeability of pectin-based composites. The WVP values decreased by 11% to 54%, depending on the pectin type and ZY concentration. A decrease in WVP of films modified with different clays was also observed by other authors and presented in the literature [26,27,28]. This behavior was attributed to the creation of multiple parallel layers, which created a tortuous path for water vapor and consequently induced a decrease in permeability. It is worth underlining that an improvement in WVP values for composite films was the following PC > PA, indicating stronger zeolite interaction with the citrus pectin than apple one. In general, the filler incorporation significantly (*p* < 0.05) reduced the water vapor permeability of pectin-based films. Among all the investigated films, the PC/ZY0.2 film revealed the highest water vapor barrier values, reaching 2.05×10^−10^ g/m s Pa. The WVP values of pectin/ZY films are comparable to the ones reported in the literature for other polysaccharide-based films [29,30,31,32,33,34,35]. On the other side, the obtained values of water vapor permeability are higher by several orders of magnitude than those reported for the commercially used plastic films like polystyrene (0.1–0.5 × 10^−12^ g/m s Pa), low density polyethylene (0.07–0.09 × 10^−12^ g/m s Pa), and high-density polyethylene (0.02–0.04 × 10^−12^ g/m s Pa) [33].

### 3.4. Mechanical Analysis

The mechanical properties of pectin-based composite films are presented in Table 2. Tensile strength values of the reference pectin films were 41 MPa and 38 MPa for PC and PA, respectively. In all cases, zeolite addition caused a remarkable improvement in tensile strength, showing significant (*p* < 0.05) differences regarding neat pectin. The most pronounced one of 66% was obtained for the PC/ZY0.2 sample. Increasing zeolite Y content facilitated interfacial interactions between the pectin and filler surface. In principal, the strength of composite materials depends on the capability of stress transfer between matrix and filler [34]. Citrus pectin established stronger interplay with zeolite Y than apple pectin, probably owing to the more linear structure. Various literature data confirmed different content and branching degree of side chains in pectin structures with different origins [6,36]. As evidenced, apple pectin contains more branched side chains in comparison with citrus ones, consequently influencing not only mechanical, but also rheological and gelling properties of various pectins [36,37,38]. Thereby, highly branched side chains of apple pectin sterically hindered interactions with ZY.

Elongation at break of composite materials may depend on the fraction volume of reinforcement and its dispersion in the polymer matrix [39]. Generally, a stiff reinforcement addition reduces elongation at break values in composite materials as filler concentrates stresses [40,41]. In all cases, an increasing filler content reduced the elongation at break and increased Young’s modulus values. This could be attributed to the formation of an immobilized pectin layer in the pectin/filler interphase, which consequently decreased the flexibility of films. This trend was also noted by other researchers for various systems: pectin/montmorillonite [31], alginate/montmorillonite [42], and starch/bentonite [43].

### 3.5. Thermogravimetric Analysis (TGA)

**Figure 3:**
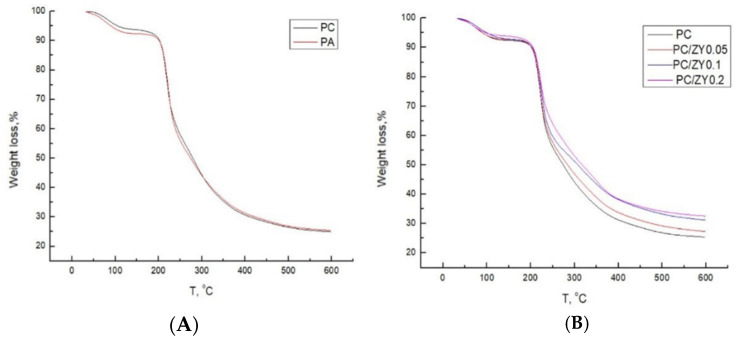
TGA curves of (**A**) pectin samples and (**B**) pectin biocomposite films.

The TGA curves and results of thermogravimetric analysis for pectin and its biocomposites are shown in Figure 3 and summarized in Table 4, respectively.

TGA curves of pure pectin films exhibited three-stage weight loss: release of free water up to 100 °C; evaporation of bound water in the range between 100 and 180 °C; and degradation of polymer chains above 180 °C. The greatest weight loss, ascribed to degradation of polymer chains, was in the range of 180 and 350 °C and reached values of 64% for PC and 65% for PA film. In this range, an intense peak related to glycosidic bonds’ degradation and a less intense one related to plasticizer degradation were observed. The addition of zeolite delayed degradation of all pectin composite films, as evidenced by lower weight loss at 350 °C, in comparison with control PC and PA films. Moreover, in all cases, the onset of main degradation step temperature (T_onset_) of composite films was slightly anticipated in comparison with the control pectin films. Although a zeolite presence caused enhanced thermal stability, a negligible influence on the peaks of the maximum degradation rate (T_degr_) was noted. The most pronounced improvement in thermal stability was observed when 0.2 wt% of the filler was added into formulation and reached values of 12.5% and 9.2% for PC and PA composite films, respectively. These results confirmed once more that the strongest interactions occurred between citrus pectin and ZY owing to the highest filler-matrix adhesion and filler dispersion within the matrix, as also evidenced by WAXD, FTIR, and mechanical tests. Well-dispersed filler may be more effective in hindering diffusion of volatile decomposition products and lead to improved thermal stability [44].

### 3.6. Applicative Potential

**Figure 4:**
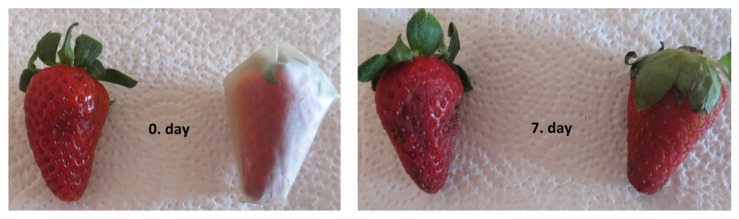
Appearance of uncoated and coated strawberry sample as a function of time.

Among all investigated samples, the citrus high methylated pectin films showed the most promising physicochemical properties. The PC/ZY0.2 film revealed the highest thermal stability and stiffness as well as moderate water vapor permeability, hence this formulation was selected for verification of food shelf-life in a proof of concept trial of 10 fruits per treatment. Samples in a form of strawberries were wrapped into PC/ZY0.2 films, thermally sealed, and stored at 10 °C. Uncoated strawberries were stored in the same conditions (see Figure 4).

After 7 days, the uncoated fruits changed their color and acquired brownish spots and mold. Weight loss of the uncoated samples was 10%. In the case of the samples wrapped in PC/ZY0.2 film, no important changes in color or mold traces were observed after 7 days. Weight loss of the wrapped strawberry samples was reduced to 8.8%. Preliminary tests proved that PC/ZY films might be a good candidate for an eco-friendly packaging of fruits with low moisture loss and low ethylene production.

## 4. Conclusions

In this study, pectin/zeolite Y composite films, produced by the solvent casting method, were investigated. Two different types of pectin were used as biopolymer matrix: citrus high methylated pectin and apple high methylated pectin. The zeolite Y content varied from 0.05 to 0.2 wt% in pectin-based composite films. An increase in zeolite content from 0.05 up to 0.2 wt% caused an enhancement in thermal stability and water vapor barrier properties. Among all investigated biopolymer matrices, citrus high methylated pectin was shown to be the most suitable for interaction with zeolite Y, which was evidenced by the highest mechanical, thermal, and water vapor barrier stability. These findings indicated the importance of the origin source of biopolymer and its chemical characteristics on the physical-chemical properties of final materials. Moreover, a filler addition may improve the functionality of biocomposite films from an application point of view. The preliminary results showed that pectin/zeolite Y film could be used to delay the decay of strawberries, in comparison with uncoated control strawberries stored at 10 °C for 7 days. Nevertheless, in order to investigate further possible applications in the food packaging sector, additional research on fungal decay, sensor, and physicochemical properties of packed fruit (weight loss, titratable acidity, and sugar solid content) is required to be performed and will be published in a forthcoming manuscript.

## Figures and Tables

**Table 1:** Physicochemical properties of Zeolite Y.

Parameter	Value
SiO_2_/Al_2_O_3_ (mol/mol)	30.3
Mean particle size (µm)	1.2
Specific surface area (m^2^/g)	710
Specific volume (cm^3^/g)	0.20

**Table 2:** Compositions of pectin-based films.

Sample	Abbreviation
Citrus pectin + PG	PC
Citrus pectin + PG + ZY 0.05 wt%	PC/ZY0.05
Citrus pectin + PG + ZY 0.1 wt%	PC/ZY0.1
Citrus pectin + PG + ZY 0.2 wt%	PC/ZY0.2
Apple pectin + PG	PA
Apple pectin + PG + ZY 0.05 wt%	PA/ZY0.05
Apple pectin + PG + ZY 0.1 wt%	PA/ZY0.1
Apple pectin + PG + ZY 0.2 wt%	PA/ZY0.2

**Table 3:** Selected mechanical parameters and WVP values of neat pectin and pectin-based films.

Sample	TS, MPa	ε, %	E, MPa	WVP × 10^−10^, g/m s Pa
PC	41 ± 1 e	15 ± 0.5 c	1340 ± 50 c	4.47 ± 0.2 d
PC/ZY0.05	45 ± 1 d	12 ± 0.5 b	1365 ± 40 c	2.98 ± 0.0.2 c
PC/ZY0.1	57 ± 2 b	12 ± 0.5 b	1426 ± 30 b	2.18 ± 0.1 a
PC/ZY0.2	68 ± 3 a	12 ± 0.5 b	1664 ± 40 a	2.05± 0.1 a
PA	38 ± 2 e	12 ± 1 b	1125 ± 70 d	4.45 ± 0.3 d
PA/ZY0.05	43 ± 2 de	10 ± 1 a	1221 ± 60 d	3.94 ± 0.2 d
PA/ZY0.1	47 ± 2 cd	10 ± 1 a	1355 ± 70 c	3.12 ± 0.1 c
PA/ZY0.2	50 ± 2 c	9 ± 1 a	1453 ± 70 b	2.70 ± 0.1 b

Treatments with different letters indicate significant differences according to Tukey’s test (*p* < 0.05).

**Table 4:** Weight loss (%) (WL%), at 100 °C, 180 °C, and 350 °C. Temperature of main degradation step (T_onset_ °C). Temperature of maximum degradation rate (T_degr_ °C).

Sample	W_L100_, %	W_L180_, %	W_L350_, %	T_onset_, °C	T_degr_, °C
PC	6	8	64	191	223
PC/ZY0.05	6	8	61	195	223
PC/ZY0.1	5	7	57	195	223
PC/ZY0.2	5	7	56	195	223
PA	5	6	65	191	219
PA/ZY0.05	4	6	63	193	220
PA/ZY0.1	4	6	62	193	220
PA/ZY0.2	5	8	59	192	221

## Data Availability

The datasets generated for this study are available on request to the corresponding author.
